# Supplementary material for: Identification of photoperiod-induced specific miRNAs in the adrenal glands of Sunite sheep (Ovis aries)
Source: Front Vet Sci. 2022 Jul 22;9:888207. doi: 10.3389/fvets.2022.888207 (PMC9354845; doi:10.3389/fvets.2022.888207)
Supplement: Supplementary file 1 [file Data_Sheet_1.ZIP › Supplementary file/miRDeep2 file.zip!/miRDeep2 file/Novel_Structure/SP42A3/Novel_574.pdf]

Provisional ID	:	NC_019459.2_4123
Score total	:	0
Score for star read(s)	:	-1.3
Score for read counts	:	-1.8
Score for mfe	:	1.6
Score for randfold	:	1.6
Score for cons. seed	:	
Total read count	:	8
Mature read count	:	8
Loop read count	:	0
Star read count	:	0

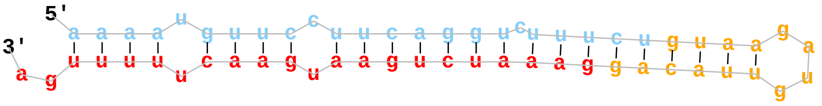

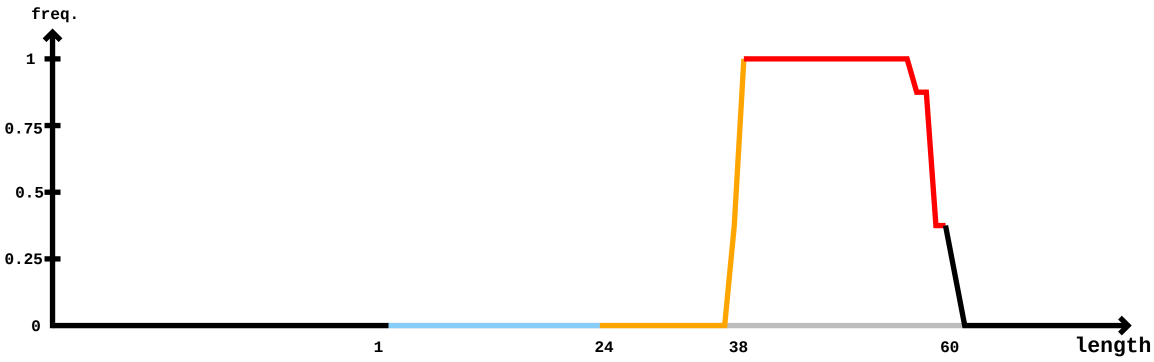

Star Mature

```
5'- cugggaagcccucauacugcuauuggguuggccaaaauguuccuucaggucuuucuguaagauguuacaggaaaucugaaugaacuuuuugaugaauccaguguuaauca   -3'    exp
    ..(((...))).........(((((((((.(.(((((.((((.(((((((.(((((((((....)))))))))))))))).)))).))))).).))))))))).......    reads      mm         sample
    .....................................................................gAaaaucugaaugaacuuu......................    1          1          seq
    .....................................................................ggaaaCcugaaugaacuuuuu....................    1          1          seq
    .....................................................................gAaaaucugaaugaacuuuuu....................    1          1          seq
    ......................................................................gaaaucugaaugaacuuuuu....................    2          0          seq
    ......................................................................gaaaCcugaaugaacuuuuuga..................    3          1          seq
```
